# Thoroughness

**Published:** 1889-08

**Authors:** Wm. H. Atkinson

**Affiliations:** New York City


### THOROUGHNESS.[1]

BY WM. H. ATKINSON. M.D., NEW YORK CITY.

Thoroughness may be taken to be the backbone of purpose when persisted in to the end of its career. But it must not be so rigidly interpreted as to preclude alacrity and flexibility in acquiring a knowledge of the exactitude of the plan of the work to enable us to discriminate the limitations and conjunctions of the parts, so as to have them harmoniously combined into a smoothly-working system, with the least possible loss of energy and the greatest increment of power. Thoroughness in preparation leads to fulness of endowment in diagnosis, prognosis, and treatment of disease. The object of thoroughness is to have work well done.

Work, to be well done, must conform to the necessities of plan as basis of purpose of the performance, of whatever sort it be. Thoroughness of preparatory knowledge makes thoroughness of effort possible, and thoroughness of effort makes possible a result thorough in its conception, preparation, and execution. Enough is contained in any modern college curriculum to eminently fit the graduate for active useful life, were it thoroughly taught and thoroughly learned. Too much effort is partly wasted in the attempt to teach large numbers. Too much stress is placed upon the personality of the instructor, and too little thoroughness is demanded of the student in taking each step as he mounts, and the wide-spread attention a large class demands precludes that individual constant notice which is vital to a comprehensive grasp by the student of the inception, progress, contemporaneous condition, and *modus operandi* of any department of human effort.

Genius needs must fly, and every advance owes genius much, if only because it points the way for the thorough worker to seek growth.

To be thorough in effort one must have been thorough in preparation. A clear conception of the conditions presenting necessarily precedes a proper or thorough ability to deal with such conditions. Thorough grasp of the work of others, whether such work was thorough or not, aids greatly in laying out effort. Thoroughness is a quality mostly of inheritance, but may also be

---

[1] Read at the semi-annual meeting of the Massachusetts Dental Society, Boston, June 6, 1889.

cultivated in those naturally negligent and loose in their modes of investigation and execution. The mass of pupils under instructors (private and in colleges) are more desirous of meeting the views of their teachers so as to pass with high marks and get their degrees than to thoroughly comprehend the teaching satisfactorily to their own understanding, which would be more effective in gaining them those high marks than any abject copying of formulæ and special interpretation, supposed to be in favor with their instructors. This simply means that the desire to pass the examinations and be recognized as in possession of knowledge is a much stronger instinct than to possess the knowledge and have recognition deferred to a later date.

The thoroughly honest mind is a sort of duplication or other self, the standards of which must be met to call forth full assent and coincidence with the soundness of the propositions brought before it for decision. This is equivalent to saying "the dentist must be born, not made."

Thoroughness is the prerequisite of rapid and permanent growth in every department of human life, and as a comprehension of the rise of dentistry—what it has been, what it is, and what we hope to make it—involves knowledge of science, art, and execution, we must possess all these and hold them in a tentative readiness to be applied whenever the occasion arises in which their application will thoroughly complete the work in hand.

As it is not the prerogative of any individual to be thus endowed, it is a great satisfaction to us to know that there is this ability somewhere among us, the correlation, aggregation, and application of which will make us adequate to the new demand, as former steps or stages of progress in our calling have so eminently proved to the benefit of mankind, by better interpretation and more thorough comprehension of the principles involved than former times have shown to be possible to men devoted even to special departments that should have fitted them for apprehension, comprehension, and practicalization of the advances of scientific attainment on a sure ratiocinative basis.

The origin of what we at present have as textual doctrine is the mass of accumulated experiment reduced to principles and set formulæ, which grew from the inspirations necessity brought to individuals.

Science scorns empiricism, and yet owes to it many facts that the inspiration of empirics led it to investigate. Old women's remedies have cured many diseases, and modern medicine is much in the dark as to the facts of the operation of drugs on the human system.

Because a certain drug has been said to be of service in a certain complication, that drug has come to be accepted as the one to be exhibited.

Any well-laid plan of procedure, thoroughly carried out by earnest and fraternal co-workers, must succeed in the ratio of the persistence with which it is pursued.

Clinical instruction was inaugurated under the sense of the need among our fraternity for its practical demonstrations. To it more than to any other single means do we owe our unprecedented advancement. Although our clinics as now held are immeasurably better than none, still much might be done to increase the benefits of such efforts.

I believe each section of a certain population should unite their local societies into a general body, and locate a permanent home in a central town where quarterly or semi-annual or more frequent meetings could be held. Such home to have connected with it a spacious laboratory and operating-rooms, the latter of which could be utilized for meetings, and the operating apparatus stored for the time being in the laboratory. These rooms to be fitted with sufficient engines, chairs, stools, stands, impression-cups, forceps, and electrical connections or batteries, furnaces, vulcanizers, lathes, vices, anvils, and rolling-mill, and to be supplied with gas, plaster, and sand,—in short, such fitting up as a properly-equipped college laboratory and operating-room would demand to the extent of the purpose in view.

This home should be accessible to members of the societies uniting in its support, and upon application to the clinic committee, or committee in charge, and to such other members of the profession as desire to give clinics before the section meeting, or any meeting held in the home. This privilege to extend over such time as might be necessary in previous preparation for the clinic, or in the case of members for experiment, or, perhaps, for regular work under control of the committee in charge, on payment of specific fees arranged by the section.

The supply of the requisite heavier tools and instruments would materially reduce the apparatus a useful demonstration requires an operator to bring, sometimes, from a great distance and at considerable expense, and too often at no pecuniary recompense to himself.

It would increase the chances for superior clinics, and elevate the tone of these demonstrations as well as increase their worth and scope.

A nice operator deserves nice surroundings, and expert work demands complete equipment of tools and instruments.

Were these reduced to such as the operator could easily carry with him, at no additional expense, many of the grave objections to public clinics would be avoided, and the quality of the work offered to clinic committees would be much advanced.

Added to this home scheme might be the establishment of a limited number of apartments for the entertainment of guests of the society; in fact, the whole scheme covers in many of its features the plant of a post-graduate school as well as a social club, and could be made to serve a very useful purpose in elevating our profession.

These sections might be combined in a union which would publish its own transactions, and could include a publication bureau for the general profession, through which notices of meetings and general professional printing and publishing could be carried on.

Many of the supplies would be willingly donated by members, and the initial expense of such a home need not be very great.

As all things must have a beginning, the effort at first might be small and cover little ground, and grow with the development of its own worth.

In some sections it might prove more feasible to offer meeting-rooms to other societies, which would tend to lower conducting expenses,—as is done by the Academy of Medicine in New York. A bureau of information would naturally result from such a home; assistants and operators could find mutual help through it; improvements in methods and apparatus could be encouraged by it, and the opportunities for exercise of beneficent action would constantly arise. The scope of such a home can hardly be named.

The clinical conference of the Massachusetts Dental Society offers a feature of value to all such meetings.

The First District Society of New York has, for a year or more past, printed an invitation to present cases for advice at its monthly clinics, which regularly appears in its notices, but the scope of the conference referred to more nearly meets my views.

I have thought to propose that society members, or others, would notify their local clinic committee of their willingness to give one or more clinics, naming the case or cases and the times when they would hold them, and that engagements could then be made, either by the committee or through it by the operator, by which not more than five dentists would be invited to witness the clinic; and as there would be several separate opportunities thus offered, preferably on regular days, as societies now hold their clinics, many would really *see* the clinic, and therefore benefit by the whole operation; whereas, now, one or two *may* see most of a clinic, but the fact of the operator working away from his accustomed surroundings hampers him, as also does the crowding of the many eager to see all they can.

Any clinic committee will agree that to secure good operators on good subjects is anything but an easy task, and a thoroughly good clinic is of immense value in bringing home to the observing dentist the daily need for thoroughness.

I do not think clinical operators receive the consideration or the credit their efforts should call forth. How many of those who observe are willing to step in and operate?

Properly started, managed, and supported, the home would be a boon indeed. The establishment of a chemical laboratory in connection with it is a further elaboration of the scheme, as also a library, museum, directory, purchasing agency, receiving office for mail and merchandise. But the opportunity is broad. Can it be embraced? Perhaps at first in part. We will see.

